# Sumoylation Influences DNA Break Repair Partly by Increasing the Solubility of a Conserved End Resection Protein

**DOI:** 10.1371/journal.pgen.1004899

**Published:** 2015-01-08

**Authors:** Prabha Sarangi, Roland Steinacher, Veronika Altmannova, Qiong Fu, Tanya T. Paull, Lumir Krejci, Matthew C. Whitby, Xiaolan Zhao

**Affiliations:** 1 Molecular Biology Program, Memorial Sloan-Kettering Cancer Center, New York, New York, United States of America; 2 Programs in Biochemistry, Cell, and Molecular Biology, Weill Cornell Graduate School of Medical Sciences, New York, New York, United States of America; 3 Department of Biochemistry, University of Oxford, Oxford, United Kingdom; 4 Department of Biology, Masaryk University, Brno, Czech Republic; 5 Howard Hughes Medical Institute, Department of Molecular Biosciences, and Institute for Cellular and Molecular Biology, University of Texas at Austin, Austin, Texas, United States of America; 6 National Centre for Biomolecular Research, Masaryk University, Brno, Czech Republic; 7 International Clinical Research Center, St. Anne's University Hospital in Brno, Brno, Czech Republic; Duke University, United States of America

## Abstract

Protein modifications regulate both DNA repair levels and pathway choice. How each modification achieves regulatory effects and how different modifications collaborate with each other are important questions to be answered. Here, we show that sumoylation regulates double-strand break repair partly by modifying the end resection factor Sae2. This modification is conserved from yeast to humans, and is induced by DNA damage. We mapped the sumoylation site of Sae2 to a single lysine in its self-association domain. Abolishing Sae2 sumoylation by mutating this lysine to arginine impaired Sae2 function in the processing and repair of multiple types of DNA breaks. We found that Sae2 sumoylation occurs independently of its phosphorylation, and the two modifications act in synergy to increase soluble forms of Sae2. We also provide evidence that sumoylation of the Sae2-binding nuclease, the Mre11-Rad50-Xrs2 complex, further increases end resection. These findings reveal a novel role for sumoylation in DNA repair by regulating the solubility of an end resection factor. They also show that collaboration between different modifications and among multiple substrates leads to a stronger biological effect.

## Author Summary

Proper repair of DNA lesions is crucial for cell growth and organism development. Both the choice and capacity of DNA repair pathways are tightly regulated in response to environmental cues and cell cycle phase. Recent work has uncovered the importance of protein modifications, such as phosphorylation and sumoylation, in this regulation. Sumoylation is known to be critical for the efficient repair of highly toxic DNA double-strand breaks in both yeast and humans, and this is partly mediated by influencing DNA end resection. However, it has been unclear for which resection factor sumoylation is important, how sumoylation influences specific attributes of the relevant targets, and how this modification is coordinated with phosphorylation-based regulation. Here, we provide exciting new insights into these issues by revealing that 1) a conserved end resection factor is a SUMO target relevant to this process, 2) this regulation favors a specific repair pathway, 3) sumoylation collaborates with phosphorylation to promote protein solubility, and 4) sumoylation influences DNA repair via an “ensemble effect” that entails simultaneous small alterations of multiple substrates. Our work reveals both a novel mechanism and a general principle for SUMO-mediated regulation of DNA repair.

## Introduction

Efficient and accurate genome repair requires regulatory mechanisms that adjust DNA repair levels and pathway usage depending on the cellular context. For example, in response to increased lesion loads, DNA repair pathways are upregulated [1]–[3]. Additionally, DNA double-strand breaks (DSBs) are repaired by either homologous recombination (HR) or non-homologous end joining (NHEJ) depending on the cell cycle stage [4], [5]. The regulatory changes in these situations occur rapidly, involve many targets, and are reversible [1]–[3]. They are often enabled by protein modifications that reversibly add modifier groups to multiple targets. The best-illustrated example of this is protein phosphorylation mediated by the DNA damage checkpoint and cyclin-dependent kinases, which occurs within minutes of changes in repair needs and affects hundreds of protein targets (e.g. [6]–[9]).

More recently, another protein modification, sumoylation, has emerged as a key regulator of genome repair (reviewed in [10]–[13]). However, many important details of how sumoylation influences DNA repair have yet to be elucidated. For example, sumoylation is important for DSB repair in humans and yeast partly by promoting DNA end resection [14]–[18]. Yet, it has been unclear for which resection factor(s) sumoylation is relevant, how sumoylation influences specific attributes of such target(s), and how this modification is coordinated with phosphorylation-based regulation.

To address these questions, we used budding yeast as a model system to examine the sumoylation of a conserved DNA end resection factor, Sae2. Sae2 collaborates with the Mre11-Rad50-Xrs2 (MRX) complex in processing multiple kinds of DSBs, including those with clean ends and ends capped with proteins or hairpin structures (reviewed in [19], [20]). Sae2 and MRX can remove the capping structure and 100–300 bp of single-stranded DNA from DSBs in a process called end clipping [21]–[29]. This first stage of DSB end resection is followed by long-range resection via parallel pathways mediated by the Exo1 exonuclease and the Sgs1/Dna2 helicase-nuclease pair [22], [24]. End clipping commits DSB repair to HR, as resected DNA ends are poor substrates for NHEJ. This commitment point is tightly regulated in conjunction with cell cycle phase [30]–[32], as NHEJ is more beneficial in G1 when sister chromatids are absent, whereas recombination constitutes more faithful repair during S and G2 phases when the synthesized sister chromatids provide accurate repair templates.

Previous studies have shown that kinases confer cell cycle-dependent regulation of end clipping [31]–[37]. Both S phase cyclin-dependent kinase (CDK) and DNA damage checkpoint kinases phosphorylate Sae2 to promote end clipping [33], [36], [37]. This is achieved at least partly by dynamically increasing Sae2 protein solubility [37]. This form of regulation is critical as Sae2 is predominantly present as inactive aggregates in G1, presumably to limit resection in this phase [36], [37]. Upon treatment with DNA damaging agents in S phase, phosphorylation of Sae2 facilitates the rapid release of active monomeric and dimeric forms from the inactive aggregates to promote end clipping, and thus HR [37].

Here, we show that the conserved sumoylation of Sae2 also promotes its functions in the processing and repair of multiple kinds of DSBs. Interestingly, like phosphorylation, sumoylation also increases the levels of soluble Sae2. We show that the two different modifications act in synergy to achieve a stronger effect on Sae2 function. Moreover, we present evidence that sumoylation of MRX also favors resection, suggesting that the coordinated sumoylation of multiple substrates leads to greater biological consequences.

## Results

### Sae2 sumoylation occurs on a single lysine in its self-association domain

**Figure 1:**
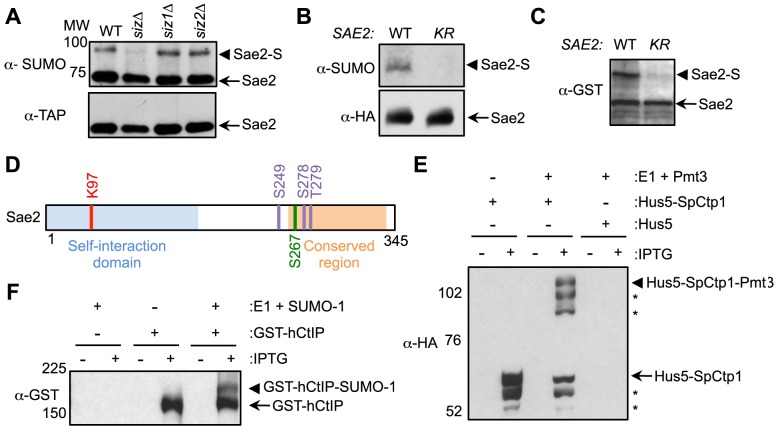
Sae2 sumoylation site mapping and conserved sumoylation of Sae2 orthologs. **A**. Sae2 sumoylation is abolished by mutating the Siz SUMO ligases. TAP-tagged Sae2 was immunoprecipitated and its sumoylated form (Sae2-S) was detected as a band migrating above the unmodified band (Sae2) by western blotting with anti-SUMO antibody in wild-type (WT), *siz1Δ*, and *siz2Δ* cells, but not in *siz1Δ siz2Δ* (*siz*) cells. Unmodified protein was detected by antibody recognizing the TAP tag. **B**. Sae2 sumoylation is abolished by the *K97R* mutation *in vivo*. HA-tagged Sae2 (WT) or Sae2-K97R (KR) expressed from its endogenous promoter was analyzed as in A. **C**. Sae2-K97R is not sumoylated in *E. coli*. GST-tagged Sae2 (WT) or Sae2-K97R (KR) co-expressed with SUMO and sumoylation enzymes was examined by western blotting with anti-GST antibody. *sae2-K97R* abolished the slower migrating form of the protein indicative of lack of sumoylation. **D**. Schematic of Sae2 showing self-interaction domain, conserved domain and modification sites. Two major Mec1 and Tel1 phosphorylation sites and adjacent site (S249, S278 and T279) are in purple, the S-CDK phosphorylation site (S267) is in green, and sumoylation site (K97) is in red. **E–F**. The fission yeast and human Sae2 orthologs, SpCtp1 (E) and hCtIP (F) respectively, can be sumoylated in *E. coli*. (E) HA-tagged SpCtp1 fused with SUMO E2 Hus5 (Hus5-SpCtp1) was co-expressed with SUMO E1 enzymes Rad31 and Fub2 (E1) and GST-tagged fission yeast SUMO (Pmt3) in *E. coli*. IPTG-induced co-expression of Hus5-SpCtp1 with SUMO and SUMO E1 resulted in the appearance of slow migrating bands above the unmodified Hus5-SpCtp1p on western blots probed with anti-HA antibody, indicative of sumoylation. (F). GST-tagged hCtIP was expressed by IPTG induction with or without the SUMO conjugating enzymes E1 and E2 and SUMO-1 in *E. coli* as indicated. The soluble protein extracts were analyzed by western blotting using anti-GST antibody.

We and others recently reported that five proteins involved in DNA end resection are sumoylated upon DNA damage in budding yeast [17], [18]. Here we examined the sumoylation of the Sae2 protein. The Sae2 sumoylated form migrates ∼20 kDa higher than the unmodified form upon SDS-PAGE, as expected from the typical up-shift caused by mono-sumoylation (Fig. 1A). The sumoylated form can be preferentially detected by an antibody against the SUMO moiety (Fig. 1A and [17]). In addition, this modification was abolished by the simultaneous removal of both the homologous Siz1 and Siz2 SUMO ligases, but not of either single ligase (Fig. 1A). These results validate Sae2 sumoylation and indicate that the Siz ligases redundantly sumoylate Sae2.

To evaluate the functional consequences of Sae2 sumoylation, we identified the lysine that is targeted for sumoylation. Sae2 possesses two sumoylation consensus motifs, ψKxE/D, where ψ is a large hydrophobic acid [38], [39]. Mutating one of these sites, K97, to arginine abolished its sumoylation *in vivo* (Fig. 1B). This residue is conserved among Sae2 orthologs in closely related *Saccharomyces* species (Fig. S1A). To confirm that K97 is the SUMO conjugation site, Sae2 was co-expressed with sumoylation enzymes in *E. coli* to enable its sumoylation (see Methods). A higher-migrating form of Sae2 in the purified protein prep was specifically eliminated by treatment with the desumoylase Ulp1, indicating that it is the sumoylated form of Sae2 (Fig. S1B). Consistent with the *in vivo* finding, Sae2-K97R mutant protein was not sumoylated *in vitro* (Fig. 1C). Together, our *in vivo* and *in vitro* data indicate that lysine 97 is the bona fide SUMO conjugation site on Sae2. K97 is located within the N terminal domain that is important for self-association in several organisms (Fig. 1D and [37], [40]–[42]).

### Sae2 orthologs are also sumoylated

As conserved modification of a protein in different organisms is indicative of functional importance, we examined whether Sae2 orthologs that share DNA resection functions are also targeted for sumoylation. To this end, we subjected recombinant Sae2 orthologs, namely fission yeast Ctp1 and human CtIP, to sumoylation in *E. coli* using reconstituted fission yeast and mammalian SUMO conjugating systems, respectively. Both SpCtp1 and hCtIP exhibited a slow migrating modified form only upon co-expression of SUMO and conjugating enzymes (Fig. 1E–1F), suggesting that they can be sumoylated. The conserved sumoylation of Sae2 orthologs supports the notion that this modification can be functionally relevant.

### Sae2 sumoylation facilitates processing of complex DSB ends

**Figure 2:**
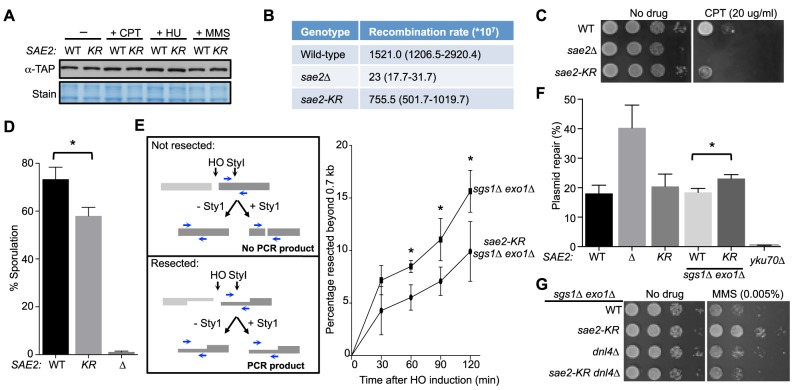
Lack of Sae2 sumoylation impairs Sae2 function. **A**. Sae2 sumoylation does not affect its protein levels. Sae2-K97R (KR) protein levels are similar to wild-type (WT) before (-) or after treatment with CPT, hydroxyurea (HU) or MMS at 30°C. Extracts from *SAE2-TAP* cells exposed to the indicated agents were analyzed by western blotting with antibody against TAP. **B**. Recombination rates at hairpin-capped DSBs. See Methods for experimental details. Median recombination rates are shown, with the range in brackets. *sae2-K97R* and *sae2Δ* exhibit moderate and strong reductions respectively in recombination rates. **C**. *sae2Δ* and *sae2-K97R* show different levels of sensitivity to CPT. 10-fold serial dilutions were used. **D**. *sae2-K97R* moderately reduces sporulation efficiency. See Methods for experimental details. The difference between wild-type and *sae2-K97R* cells is statistically significant (p<0.05, asterisk). **E**. *sae2-K97R* impairs DSB resection in *sgs1Δ exo1Δ* cells. Left: Schematic illustrating the qPCR-based resection assay. Induction of the HO endonuclease results in a double-strand break at the *MAT*a locus. The fate of the fragment to the right of the HO cut (dark grey) can be followed by PCR. The unresected, StyI-digested DNA does not yield PCR product using the indicated primer pair (blue arrows), whereas undigested or resected DNA does. Right: The percentage of resected fragment was calculated by the formula detailed in Methods, which compares the PCR yields of digested and mock-digested DNA normalized to amplification at a control locus. For each strain, values from at least three experiments were averaged and standard deviations were calculated. The difference between the two genotypes at indicated time points is statistically significant (p<0.05, asterisk). **F**. Plasmid-based NHEJ is increased by *sae2-K97R* in *sgs1Δ exo1Δ* cells. Cells were transformed with either BamHI-digested or undigested plasmid DNA and plated on media selective for the plasmid. Percentage plasmid repair was calculated by dividing the number of colonies recovered from digested samples with undigested. See Methods for experimental details. For each genotype, values from at least three experiments were averaged and standard deviations were calculated. Asterisk indicates statistically significant difference (p<0.05). **G**. *sae2-KR* suppresses the MMS sensitivity of *sgs1Δ exo1Δ* cells in a Dnl4-dependent manner. 10-fold serial dilutions were used.

Next, we investigated whether abolition of Sae2 sumoylation affects its functions by studying the *sae2-K97R* allele. *sae2-K97R* did not affect Sae2 protein levels in normal growth conditions or after genotoxin treatment at either 30°C or 37°C, excluding an effect of sumoylation on general protein stability (Fig. 2A and S1C Fig.). We then tested whether *sae2-K97R* affects the processing and repair of complex DNA ends, such as those capped by hairpin structures or covalently linked with proteins, using established assays. To query hairpin repair *in vivo*, we measured recombination that requires removal of hairpins formed through inverted Alu sequences at DSBs [27], [43]. Consistent with a previous report, deleting *SAE2* greatly reduced the recombination rate measured in this assay (Fig. 2B and [27], [43]). *sae2-K97R* showed a 2-fold reduction (Fig. 2B), suggesting a moderate deficiency of Sae2 function in hairpin removal.

To examine processing of DSB ends that are covalently linked with proteins, we first examined Sae2-mediated processing of DSBs capped with Top1, which are induced upon camptothecin (CPT) treatment [29], [44]. Consistent with previous reports, *sae2*Δ cells were sensitive to CPT (Fig. 2C and [29], [44]). *sae2-K97R* cells exhibited an increase in sensitivity to CPT at 37°C (Fig. 2C), suggesting that sumoylation of Sae2 contributes to CPT repair. Consistent with this, Sae2 sumoylation is induced by CPT treatment and elevated temperature (S1D Fig.). Second, we examined sporulation efficiency, as Sae2-mediated removal of Spo11 conjugated to DSB ends in meiosis is required for sporulation (Fig. 2D and [28], [45]–[47]). *sae2-K97R* homozygous diploid cells exhibited a reproducible 20% reduction in this assay, indicating a moderate defect (Fig. 2D, p<0.05). Taken together, these results show that *sae2-K97R* is partially defective in the processing and repair of complex DSBs.

### Sae2 sumoylation promotes DNA end clipping

We proceeded to assess whether Sae2-mediated end clipping of clean DSB ends is affected by *sae2-K97R*. In yeast, end clipping can be directly assayed at DSBs induced by the endonuclease HO at the *MAT* locus [22]. As shown previously, because end clipping is an intermediate stage in end resection, it can be best monitored when the downstream extensive resection step is blocked by removing Sgs1 and Exo1 [22]. Both qPCR- and Southern blot-based assays can be used to assess Sae2 function in this setting. The two assays take advantage of the fact that single-stranded DNA generated by resection is resistant to restriction enzyme digestion. In the qPCR-based assay, PCR products amplified using primers flanking a StyI site located 700 bp distal to the DSB are compared between digested and undigested samples (Fig. 2E, left panel, and [24], [30], [48]). PCR products from a control locus, *ADH1*, are used for normalization (see Methods). Using this assay, we found that *sae2-K97R* exhibited 60–80% of the wild-type level of resection in a time course of 120 minutes in the *sgs1*Δ* exo1*Δ background (Fig. 2E, right). The lethality of *sae2*Δ* sgs1*Δ* exo1*Δ prevents comparison of *sae2-K97R* defects with *sae2*Δ in this setting [22].

In the Southern blot assay, end clipping products run as a smear of bands below the HO cut (unprocessed) fragment, and both types of bands are detected by a radio-labeled probe specific to a sequence flanking the DSB (S2A Fig. and [22]). In *sgs1*Δ* exo1*Δ cells, the intensity of the smear moderately increased as the unprocessed fragment diminished with time (S2A–S2C Fig.), signifying the progress of end clipping [22]. Introducing the *sae2-K97R* mutation reduced end clipping efficiency, as seen by the persistence of the unprocessed fragment and decreased intensity of the smear below (S2A–S2C Fig.). Quantification of three independent strains indicated a reduction of up to 50% in the fraction of end clipping products among total cut fragments (Fig. S2C). Taken together, both the qPCR- and Southern blot-based assays show that lack of Sae2 sumoylation impairs end clipping of clean DSBs.

### Sae2 sumoylation suppresses NHEJ in the *sgs1*Δ* exo1*Δ background

As end clipping disfavors NHEJ, its impairment would promote NHEJ [36], [49], [50]. Accordingly, a prediction of the observed end clipping defect in *sae2-K97R sgs1*Δ* exo1*Δ cells compared with *sgs1*Δ* exo1*Δ cells (Fig. 2E and S2A–S2C Figs.) is that the former should have higher NHEJ levels. Indeed, we detected an ∼30% increase in NHEJ in the triple mutant compared with the double, using a standard chromosomal NHEJ assay (S2D Fig.). As this assay was performed side-by-side with the Southern blot-based resection assay, equal efficiency of HO cleavage between genotypes was confirmed (S2A Fig.). We also used a well-established plasmid-based NHEJ assay in which cells are transformed with linearized or undigested plasmid DNA, and survival on selective media serves as a readout for successful repair by NHEJ [51]. *sae2-K97R* again exhibited a moderate increase in this assay in the *sgs1*Δ* exo1*Δ* background, while its effect in the SGS1 EXO1 background was not statistically significant (Fig. 2F).*

We noticed that *sae2-K97R sgs1*Δ* exo1*Δ showed more resistance to the DNA damaging agent methyl methanesulfonate (MMS) than *sgs1*Δ* exo1*Δ (Fig. 2G). This is in contrast to the inviability of *sae2*Δ* sgs1*Δ* exo1*Δ [22]. One interpretation is that moderate reduction of end clipping in the absence of extensive resection allows more NHEJ, thus better survival, whereas complete loss of resection confers lethality even with endogenous levels of DNA damage. Supporting this idea, the higher MMS resistance of *sae2-K97R sgs1*Δ* exo1*Δ depends on the NHEJ factor Dnl4 (Fig. 2G). These findings and the increased NHEJ seen for *sae2-K97R* are consistent with this mutant's impairment in end resection (Fig. 2E and S2A–S2C Fig.).

### Sumoylation and phosphorylation of Sae2 contribute separately to DNA damage resistance

**Figure 3:**
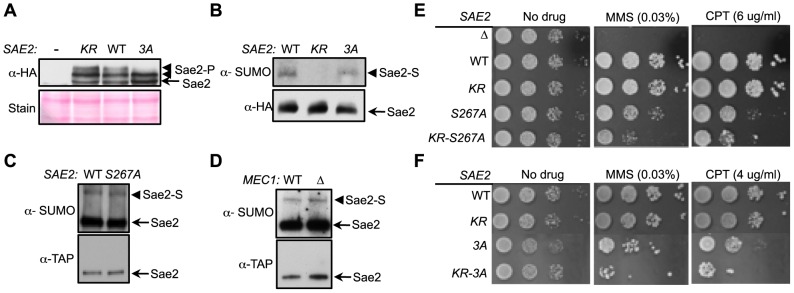
Sumoylation and phosphorylation of Sae2 occur independently and make separate contributions to DNA damage resistance. **A**. Phosphorylation of Sae2 is unaffected by lack of its sumoylation. Phosphorylation of HA-tagged Sae2 after MMS treatment was examined in the indicated strains. *sae2-3A* (*3A*), but not *sae2-K97R* (*KR*), abolishes one form of phosphorylated Sae2 (Sae2-P). **B–D**. Sumoylation level of Sae2 is not affected by lack of Sae2 phosphorylation or the Mec1 kinase. (B). Mutating major Mec1/Tel1 phosphorylation sites does not affect Sae2 sumoylation. (C). Sae2 sumoylation level in Sae2-S267A mutant defective in S-CDK phosphorylation is comparable to wild-type. (D). Deletion of Mec1 does not affect Sae2 sumoylation. Experiments were performed as in Fig. 1A. **E–F**. Combining mutations of Sae2's sumoylation site and phosphorylation sites results in additivity in CPT and MMS sensitivities. Indicated strains were examined for growth on normal media and media containing either MMS or CPT. 10-fold serial dilutions were used.

As phosphorylation of Sae2 is required for its resection function and DNA damage resistance [33], [36], [37], we asked whether *sae2-K97R* interferes with this modification. The phosphorylated forms of Sae2 manifest as slower migrating bands, and mutating two main Mec1/Tel1 phosphorylation sites and an adjacent serine, namely S249, S278 and T279 (*sae2-3A*, [33], [37]) results in the loss of the top bands (Figs. 1D and 3A). We found that Sae2-K97R exhibited a similar mobility shift as its wild-type counterpart (Fig. 3A), suggesting that sumoylation of Sae2 does not interfere with its phosphorylation.

To further elucidate the interplay between the two modifications, we assayed the sumoylation levels of Sae2 phosphorylation mutants. Neither *sae2-3A* nor *sae2-S267A*, which abrogates CDK-mediated phosphorylation, affected Sae2 sumoylation (Fig. 3B–3C). Consistent with this, *mec1*Δ cells exhibited normal levels of Sae2 sumoylation (Fig. 3D), despite being deficient for Sae2 phosphorylation [33]. Together, these results show that sumoylation of Sae2 does not require its phosphorylation.

As phosphorylation and sumoylation of Sae2 occur independently, and both contribute to Sae2 function, we examined whether their biological effects are additive. We found that combining the *K97R* and *3A* mutations, or the *K97R* and *S267A* mutations, resulted in greater sensitivity to MMS and CPT compared to mutants that were defective for only one modification (Fig. 3E–3F). These results indicate that sumoylation and phosphorylation of Sae2 make separate contributions to DNA damage resistance.

### Sumoylation of Sae2 increases the levels of soluble Sae2

We proceeded to examine how sumoylation of Sae2 affects its function. As shown recently, an important means of regulating Sae2 by protein modification is through increasing its solubility [37]. Sae2 is primarily in inactive aggregate forms in G1, whereas the soluble and active fraction of Sae2 increases upon entering S phase in the presence of DNA damage [37]. Phosphorylation of Sae2 by Mec1 and S-CDK promotes this increase [37]. Considering the genetic interaction between the two types of Sae2 modifications, we examined if sumoylation also alters the levels of soluble Sae2.

**Figure 4:**
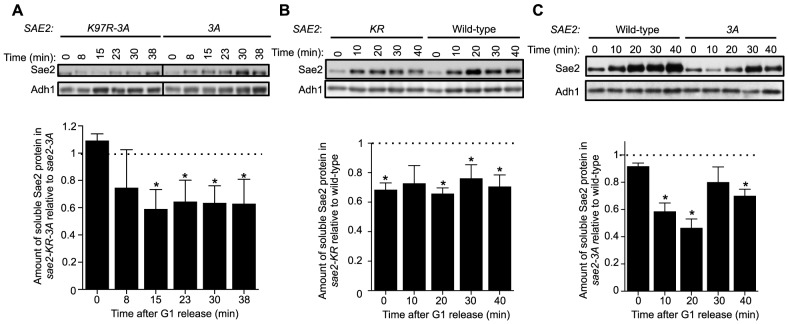
Sumoylation of Sae2 increases the levels of soluble Sae2. G1-arrested cells were released into S phase in the presence of 0.03% MMS and cell extracts at indicated time points after release were prepared. Soluble fractions of Sae2-HA from indicated strains were examined by western blots using anti-HA antibody and anti-Adh1 antibody (loading control). Representative results are shown on top, and quantification of the relative amount of soluble Sae2 between the two compared strains from at least three independent trials is shown at the bottom. Asterisks indicate statistically significant differences (p<0.05). **A**. The level of soluble forms of Sae2 is decreased in *sae2-K97R-3A* cells compared to *sae2-3A*. **B**. The level of soluble forms of Sae2 is decreased in *sae2-K97R* cells compared to wild-type. **C**. The level of soluble forms of Sae2 is decreased in *sae2-3A* cells compared to wild-type.

Using an established solubility assay, we examined G1-arrested cells after release into 0.03% MMS [37]. Consistent with our genetic data, levels of soluble Sae2-K97R-3A protein were significantly lower than that of Sae2-3A after cells were released from G1 (Fig. 4A). We also observed a similar but smaller decrease in levels of soluble Sae2-K97R when compared with that of wild-type Sae2 (Fig. 4B). In this case, the level of soluble Sae2-K97R protein is decreased by ∼25% compared to wild-type in a cell cycle-independent manner, suggesting that sumoylation by itself also affects Sae2 solubility. This reduction is less severe than that of Sae2-3A alone, which showed an S phase-specific decrease in Sae2 soluble levels by up to 50%, compared with wild-type Sae2 (Fig. 4C). These results suggest that while both phosphorylation and sumoylation promote Sae2 solubility, the former has a stronger effect. As the solubility difference between Sae2-3A and Sae2-K97R mutants is only 25%, yet their MMS sensitivities differ greatly, it is possible that the Sae2-3A has additional defects besides solubility.

### Evidence supporting a role for MRX sumoylation in DSB processing

Because *sae2-K97R* exhibited mild resection defects and genotoxin sensitivity (Fig. 2 and S2 Fig.), and not to the level that has been reported for SUMO E2 (e.g. [17]), we reasoned that sumoylation likely wields a strong influence on this process by additionally targeting other factor(s), such as MRX. As mapping sumoylation sites on the three subunits of MRX proved difficult, we devised a genetic strategy to reduce MRX sumoylation. It has been shown that the catalytic domain of the de-sumoylating enzyme Ulp1 (UD) when fused with a protein can lead to the targeted removal of SUMO conjugated to the protein or its interactors [52]. A fusion with mutations of four residues required for catalytic activity and SUMO interaction (UD*) was used to control for the effect of tagging with this domain [52].

**Figure 5:**
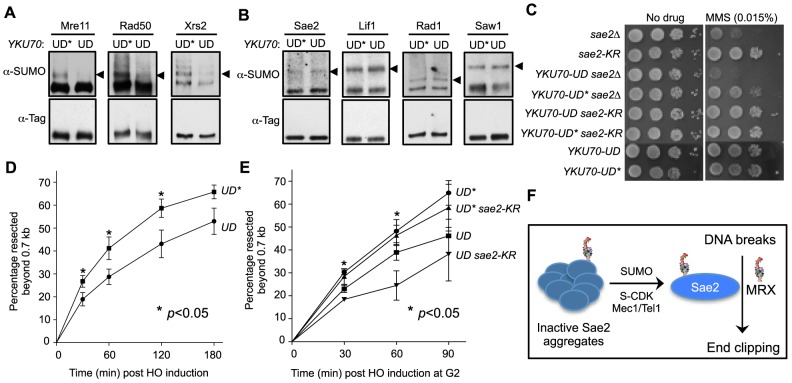
Sumoylation of MRX contributes to DNA end resection. **A–B**. Sumoylation of the three MRX subunits (Mre11, Rad50 and Xrs2), but not that of Sae2, Lif1, Rad1 or Saw1, is decreased in *YKU70-UD* cells compared to YKU70-UD*. Experiments were done as in Fig. 1A, and cells were treated with MMS. Triangles indicate the mono-sumoylated forms of the proteins examined. **C**. *YKU70-UD*, but not YKU70-UD*, sensitizes *sae2Δ* to MMS. 10-fold serial dilutions were used. **D**. Resection is impaired in *YKU70-UD* compared to YKU70-UD*. qPCR-based resection assay was performed as described in Fig. 2E. At least three spore clones for each genotype were tested. Asterisks indicate statistically significant differences (p<0.05). **E**. *sae2-K97R* impairs resection in *YKU70-UD*, but not YKU70-UD*, cells. Assay was performed as in Fig. 2E. At least three spore clones for each genotype were tested. Asterisks indicate statistically significant differences between *YKU70-UD* and *YKU70-UD sae2-KR* (p<0.05). Note that the values for *YKU70-UD* and YKU70-UD* are significantly different at all time points (p<0.05). **F**. Working model for the role of Sae2 sumoylation in DSB resection. MRX denotes the Mre11-Rad50-Xrs2 complex.

MRX physically interacts with the Ku complex, which arrives at DSBs concomitantly with MRX [53], [54]. We tested if fusing the UD domain to the Ku70 subunit (*YKU70-UD*) can specifically decrease MRX sumoylation. To this end, we introduced the *YKU70-UD* or YKU70-UD* constructs at the endogenous *YKU70* locus with its native promoter. As shown in Fig. 5A, sumoylation of all three subunits of MRX was either abolished or strongly reduced in cells expressing *YKU70-UD* compared with YKU70-UD* control cells. To assess the specificity of desumoylation, we examined proteins that arrive at DSBs around the same time as MRX [53], [55]. *YKU70-UD* did not affect the sumoylation of Sae2, or the Ku-interacting protein Lif1 (Fig. 5B). In addition, sumoylation of the downstream recombination proteins Rad1 and Saw1 was not affected by *YKU70-UD* (Fig. 5B). Together, these results suggest that *YKU70-UD* can limit MRX sumoylation with good specificity.

We then examined whether *YKU70-UD* has a phenotype indicative of defective MRX-mediated resection. As MRX deficiency can exacerbate *sae2*Δ sensitivity to DNA damaging agents in certain contexts [44], we tested whether reducing MRX sumoylation by *YKU70-UD* causes a similar phenotype. Indeed, we found that *YKU70-UD* worsened the MMS sensitivity of *sae2*Δ cells, while YKU70-UD* conferred suppression (Fig. 5C). The latter effect is likely due to the tag's interference with Ku function, such as in inhibiting HR by Exo1 exclusion [44], [56], [57]. That *YKU70-UD* is additive with *sae2*Δ suggests that the defects caused by reduced MRX sumoylation overrides any suppression conferred by defective Ku function. This in turn suggests the possibility that reduced MRX sumoylation impairs its resection function. To test this idea, we examined resection dynamics in *YKU70-UD* vs. YKU70-UD* cells. In both qPCR- and Southern blot-based assays, DSB resection was decreased by 15–20% in *YKU70-UD* cells compared to YKU70-UD*, most obviously at early time points (Fig. 5D–5E and S3A–S3B Fig.), suggesting that sumoylation of MRX facilitates resection.

To assess if the sumoylation of MRX and Sae2 independently promotes resection, we measured end resection in *YKU70-UD sae2-K97R* cells. As shown in Fig. 5E, *sae2-K97R* further compromised resection in *YKU70-UD*, but not YKU70-UD*, cells. The moderate additivity in resection defects did not result in exacerbation of *YKU70-UD*'s MMS sensitivity by *sae2-K97R* (Fig. 5C), likely because it is insufficient to confer MMS sensitivity or *YKU70-UD* exerts compensatory effects due to impaired Ku function. Taken together, our results suggest that sumoylation of MRX, in addition to that of Sae2, contributes to resection.

## Discussion

Regulation of DNA repair pathway levels and capacity in response to cell cycle changes and lesion loads is important for genome maintenance and damage resistance. Despite recent progress, many forms and targets of regulation are not yet identified or understood. One of the most highly regulated DNA repair proteins is the end resection factor, Sae2. Its solubility is tightly controlled such that its activity and other functions are limited in G1 phase and increased in S phase under damage conditions [37]. We reveal here that regulation of Sae2 solubility is partly mediated by sumoylation. We also show that sumoylation collaborates with checkpoint-dependent phosphorylation in facilitating Sae2 function in end clipping. Furthermore, we provide evidence that sumoylation promotes DNA end resection by additionally targeting the MRX nuclease. This work reveals a novel mechanism of SUMO-mediated regulation of DNA repair and uncovers an example wherein sumoylation and phosphorylation, as well as multiple sumoylation events, collaborate to promote nuclease function (see model in Fig. 5F).

Proteins that cleave DNA are double-edged swords, and unscheduled DNA cleavage has to be minimized. In general, upstream constraints such as limiting recruitment to lesions can restrict the activity of downstream factors (e.g. [58]–[61]). As Sae2 is one of the first resection proteins to arrive at DSBs without any known recruiters [55], it is not surprising that it is subjected to other forms of regulation. Our findings and previous reports strongly suggest that Sae2 regulation can be achieved by different protein modifications that collaborate to ensure timely availability of the active forms of the protein [37], [62], [63].

Thus far, regulation of protein solubility by sumoylation has been reported only for proteins involved in neuronal diseases (e.g. [64]–[66]). We now provide the first example wherein sumoylation regulates the solubility of a DNA metabolism protein. As protein aggregation is a widespread phenomenon caused by high intrinsic aggregation potential of the protein (e.g. [67], [68]), it is conceivable that SUMO-mediated protein solubilization is a general effect. Such an effect by SUMO may be similar to its promotion of solubility in recombinant protein applications [69], [70]. Thus, in addition to the previously proposed glue effect of sumoylation in bridging interactions in complexes [18], [71], [72], sumoylation can also have the opposite effect of “anti-glue” to disperse proteins from aggregates. As sumoylation occurs in the Sae2 self-association domain and at a region of high aggregation potential (S4A Fig. and [41], [73]), steric or charge changes conferred by sumoylation in these regions can disfavor aggregation.

Several independent analyses show that lack of Sae2 sumoylation moderately reduces end resection and increases NHEJ (Fig. 2B–2F and S2A–S2D Fig.). The correlation of these effects with changes in the levels of soluble Sae2 suggests that the decreased availability of active Sae2 can at least partly account for the end resection defects and NHEJ increase, though other possibilities such as defective DSB recruitment cannot be excluded. As the resection defect of *sae2-K97R* is less severe than that of the SUMO E2 mutant, sumoylation of additional resection factors also likely matters. Indeed, reduction of MRX sumoylation also impairs resection, and in a manner additive with *sae2-K97R* (Fig. 5). Although a thorough examination of MRX sumoylation awaits mapping of sumoylation sites on all three subunits, these results suggest that sumoylation achieves a large biological effect by simultaneously inducing small changes in multiple substrates (“ensemble effect”). This suggestion is consistent with the observations that several dozen repair proteins are sumoylated upon DNA damage [17], [18], [74], and individual non-sumoylatable mutants usually exhibit only mild phenotypes (e.g. [75]–[77]). We propose that the ensemble effect model is common in DNA repair regulation and other processes to confer robustness to a system. We also note that the usefulness of this strategy is also seen for other protein modifications (e.g. [32], [62]), and that as in the case of sumoylation, the effects of a particular modification are unique to the substrate, rather than conforming to a general mechanism (e.g. [8], [78]–[80]).

In summary, our work provides strong evidence for a new role for sumoylation in regulating DNA repair and its collaboration with phosphorylation-based regulation. Considering that only a few sumoylated substrates in DNA repair have been examined in detail thus far, future studies on additional substrates and the interplay between sumoylation and other forms of regulation will greatly expand our knowledge of how repair pathway levels and choice are determined in cells.

## Materials and Methods

### Yeast strains and genetic manipulations

Strains used are listed in Table 1. Only one strain per genotype is shown for simplicity, but at least two strains per genotype were tested in each assay. Standard yeast protocols were used for strain generation, growth and medium preparation. As *siz1*Δ* siz2*Δ results in amplification of the 2 micron plasmid [81], strains with *siz1*Δ* siz2*Δ mutations were cured of the plasmid as described [82].

**Table 1:** Yeast strains used in the study.

Strain	*Genotype*	Source
W1588-4A	*MATalpha leu2-3,112 ade2-1 can1-100 his3-11,15 ura3-1 trp1-1 RAD5*	R. Rothstein
T587	*MATa SAE2-TAP::HIS3*	This study
X4086-10B	*SAE2-TAP::HIS3 mms21-11::HIS3*	This study
X6231-9B	*SAE2-TAP::HIS3 siz1Δ::KAN*	This study
X6231-2A	*SAE2-TAP::HIS3 siz2Δ::URA3*	This study
X3200-1C	*sae2-K97R-TAP::HIS3*	This study
X4088-81B	*MATa leu2::GAL1-HO-LEU2 hmrΔ hmlΔ sgs1Δ::HYG exo1Δ::KAN*	This study
X3868-25C	*MATa leu2::GAL1-HO-LEU2 hmrΔ hmlΔ sae2-K97R sgs1Δ::HYG exo1Δ::KAN*	This study
Z358-3	*MATalpha sae2-K97R*	This study
X3293-1B	*sae2Δ::KAN*	This study
T954-1	*sae2-S267A-TAP::HIS3*	This study
T1237-2	*sae2-K97R-3HA::URA3*	This study
T1240-1	*sae2-S249A, S278A, T279A-3HA::URA3*	This study
X1259-1A	*SAE2-TAP::HIS3 (S288C)*	This study
X1382-7B	*SAE2-TAP::HIS3 mec1Δ::HYG sml1Δ::KAN (S288C)*	This study
T1184-5	*sae2-S267A::KAN*	This study
T1185-3	*sae2-K97R, S267A::KAN*	This study
T1165-18	*sae2-S249A, S278A, T279A::KAN*	This study
T1166-15	*sae2-K97R, S249A, S278A, T279A::KAN*	This study
T1241-10	*MATa sae2-K97R, S249A, S278A, T279A-3HA::URA3*	This study
G786	*MATa SAE2-3HA::URA3*	J. Petrini
T1238-2	*MATalpha sae2-S267A-3HA::URA3*	This study
T1239-29	*MATalpha sae2-K97R, S267A-3HA::URA3*	This study
X6006-1B	YKU70-V5-UD*::KAN MRE11-TAP::HIS3	This study
X6005-2D	*YKU70-V5-UD::KAN MRE11-TAP::HIS3*	This study
X6025-3A	YKU70-V5-UD*::KAN RAD50-3HA::HIS3	This study
X6024-2A	*YKU70-V5-UD::KAN RAD50-3HA::HIS3*	This study
X6039-3C	YKU70-V5-UD*::KAN XRS2-TAP::HIS3	This study
X6038-2C	*YKU70-V5-UD::KAN XRS2-TAP::HIS3*	This study
X6023-1B	YKU70-V5-UD*::KAN SAE2-TAP::HIS3	This study
X6022-4A	*YKU70-V5-UD::KAN SAE2-TAP::HIS3*	This study
X6112-1D	YKU70-V5-UD*::KAN LIF1-TAP::HIS3	This study
X6111-2C	*YKU70-V5-UD::KAN LIF1-TAP::HIS3*	This study
X6057-9C	YKU70-V5-UD*::KAN RAD1-TAP::HIS3 SAW1-TAP::HIS3	This study
X6056-5B	*YKU70-V5-UD::KAN RAD1-TAP::HIS3 SAW1-TAP::HIS3*	This study
T1653-12D	*YKU70-V5-UD::KAN*	This study
T1655-4D	YKU70-V5-UD*::KAN	This study
X5945-4C	*YKU70-V5-UD::KAN sae2Δ::HYG*	This study
X5947-12D	YKU70-V5-UD*::KAN sae2Δ::HYG	This study
X6040-1A	*YKU70-V5-UD::KAN sae2-K97R*	This study
X6041-2C	YKU70-V5-UD*::KAN sae2-K97R	This study
X6482-9A	*MATa ade3::GAL1-HO hmrΔ hmlΔ YKU70-V5-UD::KAN*	This study
X6483-9A	MATa ade3::GAL1-HO hmrΔ hmlΔ YKU70-V5-UD*::KAN	This study
X6480-5D	*MATalpha ade3::GAL1-HO hmrΔ hmlΔ YKU70-V5-UD::KAN sae2-K97R*	This study
X6481-41B	MATa ade3::GAL1-HO hmrΔ hmlΔ YKU70-V5-UD*::KAN sae2-K97R	This study
G938	*MATa ade5-1, his7-2, leu2-3,112:: p305L3 (LEU2), trp1-289, ura3-D, lys2::AluIR in CGL*	K. Lobachev
G939	*G938 sae2Δ::HgrB in CGL*	K. Lobachev
T1772-1	*G938 sae2-K97R::KAN in CGL*	This study
G784	*MATa/MATalpha, ho::LYS2, lys2, leu2, arg4 in SK1*	S. Keeney
X6556-1	*MATa/MATalpha, ho::LYS2, lys2, leu2, arg4, sae2Δ::LEU2 in SK1*	This study
X6557-1	*MATa/MATalpha, ho::LYS2, lys2, leu2, arg4, sae2-K97R::KAN in SK1*	This study
X4217-6C	*sgs1Δ::HIS exo1Δ::KAN*	This study
X4217-6D	*sgs1Δ::HIS exo1Δ::KAN sae2-K97R*	This study
X6484-43B	*sgs1Δ::HIS exo1Δ::KAN dnl4Δ::URA3*	This study
X6485-1D	*sgs1Δ::HIS exo1Δ::KAN dnl4Δ::URA3 sae2-K97R*	This study

Strains in this study are derivatives of W1588-4C, a *RAD5* derivative of W303 [88], unless indicated otherwise. All strains were constructed in this study.

### Protein preparation and detection of sumoylated proteins

Detection of the sumoylated form of Sae2 was performed as described previously [17]. In brief, log phase cultures were treated with 0.3% methyl methanesulfonate (MMS, Sigma-Aldrich) or 50 ug/ml camptothecin (CPT, Sigma-Aldrich) or at 37°C for 2 h. Cells were lysed by bead beating under denaturing conditions, and TAP-or HA-tagged proteins were immunoprecipitated. These were then washed and eluted with loading dye, followed by SDS-PAGE and western blotting with antibodies against SUMO [83], the protein A portion of the TAP tag (Sigma-Aldrich) or HA (12CA5). We note that as the F_c_ portion of the SUMO antibody interacts with the Protein A part of TAP, it also detects the unmodified protein, but more strongly so for the sumoylated form because of additional high affinity for SUMO. Protein preparation for detecting Sae2 phosphorylation and protein levels was performed as described [33]; DNA damage treatment was performed as above.

### Assessment of soluble Sae2 protein levels

Assay was performed essentially as described [37] except that all Sae2 constructs were expressed from its own chromosomal locus. In brief, G1-arrested cells were released into 0.03% MMS and samples were harvested at the indicated time points for protein and FACS analyses. Upon complete cell lysis by bead beating and removal of DNA by DNaseI treatment, cell extract was centrifuged at high speed (14k rpm for 30 min) to separate the soluble fraction from the insoluble. The soluble fractions were analyzed by SDS-PAGE and western blotting against the tag. The housekeeping protein Adh1 was used as loading control as its levels are invariant during the time course. FACS analyses show proper arrest and release for all the strains examined. We note that Sae2 is sumoylated in both G1 and S phases during this procedure (S4B Fig.). To assess Sae2 soluble forms in different strains, two spore clones of each genotype were examined in at least two independent tests. For quantification, we compared solubility between the two strains for each time point. In brief, we first determined the soluble Sae2 protein level relative to loading control for each strain at each time point, and then calculated the ratio between the genotypes to represent it in Fig. 4. The student's t test statistical analysis was performed for “Sae2 protein level relative to loading control” between the two genotypes from 6 repeats (2 trials with 3 spores).

### *In vitro* sumoylation assay

Both GST-tagged Sae2 and hCtIP were sumoylated in *E. coli* by co-expression with E1 (Aos1-Uba2), E2 (Ubc9) and SUMO-1 [84] (the pT-E1E2S1 plasmid was a gift from Dr. Hisato Saitoh). Plasmids for expression of GST-tagged Sae2 and hCtIP are derivatives of pGEX-4T1 and were a gift from Dr. Stephen Jackson [36], [85]. The plasmid expressing GST-Sae2K97R (pRS72) was made by site-directed mutagenesis of pGEX-4T1-Sae2. Plasmids were transformed into BL21 (DE3) cells, and single colonies were used to inoculate overnight cultures of LB (plus 100 ug/ml ampicillin or 25 ug/ml ampicillin and 17 ug/ml chloramphenicol), which were incubated at 30°C with shaking at 250 rpm. These starter cultures were used to inoculate fresh LB (with ampicillin plus chloramphenicol added as required) cultures to an OD_600_ of 0.1, which were then grown at 30°C with shaking at 250 rpm to an OD_600_ of 1.2. The temperature was then lowered to 25°C and 250 uM IPTG added to induce expression of the proteins. The cultures were incubated for another 16 h at 25°C before harvesting by centrifugation. The cell pellet was resuspended in PBS (pH 7.3) supplemented with 1 mM PMSF and 5 mM DTT. After sonication and centrifugation (47000×g at 4°C for 20 min), the soluble protein fraction was loaded onto an equilibrated Glutathione Sepharose Fast Flow column (GE Healthcare Life Sciences). The column was washed with 10 column volumes of PBS and the GST-Sae2 and GST-Sae2-SUMO proteins eluted with 2 column volumes of 50 mM Tris-HCl (pH 8.0), 30 mM reduced glutathione. Proteins were dialyzed against 20 mM Tris, pH 8.0 containing 150 mM NaCl at 4°C. To perform SUMO cleavage reactions, recombinant purified Ulp1 (10 nM) [86] was added to 5 uM partially purified GST-Sae2/GST-Sae2-SUMO and incubated at 23°C for 30 min in buffer containing 25 mM Tris-HCl (pH 8.0), 150 mM NaCl, 0.1% Tween-20, and 2 mM DTT. The proteins were separated on a 10% SDS PAGE gel and analyzed on a western blot probed with an anti-GST antibody (GE Healthcare Life Sciences). 3HA-tagged SpCtp1 was sumoylated in *E. coli* BL21 (DE3) cells by co-expression with the *S. pombe* E1 (Rad31+Fub2), E2 (Hus5 fused to a 6×His-tag) and SUMO (Pmt3GG). The 6×His-Hus5 is fused to 3HA-SpCtp1 and Pmt3GG is tagged with GST. Full details of the plasmid constructs will be provided elsewhere. Transformed BL21 (DE3) cells were cultured and proteins were isolated as described above.

### Inverted Alu recombination assay

These were performed as described [43]. Single colonies were picked from streakouts and allowed to grow for 3 days. Each single colony was resuspended in 0.25 ml water by vortexing (0^th^ dilution) and ten-fold serial dilutions were prepared. 100 ul of the 5^th^ dilution was plated onto complete medium, while 100 ul of the 2^nd^ dilution (or 0^th^ dilution for *sae2*Δ cells) was plated onto medium lacking lysine. Successful recombination by processing Alu-generated hairpin DSBs generates *LYS*+ colonies. Fourteen colonies were analyzed in this manner for each genotype, and the recombination rate was calculated by fluctuation analysis.

### DSB resection assays

Both qPCR- and Southern blot-based assays were performed as described [17], [48]. For both assays, a DSB at the *MAT* locus was introduced by galactose-induced expression of the HO endonuclease throughout the time course either in asynchronous (Figs. 2E and 5D, S2A–S2C and S3A–S3B Figs.), or G2-arrested cultures (Fig. 5E). Samples were collected at the indicated time points. Genomic DNA was isolated and an aliquot was subjected to digestion with XbaI and StyI. For Southern blot-based method, digested DNA was subjected to native agarose gel electrophoresis, transferred to Hybond XL (GE Healthcare) membranes, and hybridized with radiolabeled DNA probes. Quantification of intensities of bands on the Southern blots was done using ImageGauge. DSB end resection at each time point was calculated as the ratio of the signal intensity at that time point to that at the first time point after HO induction. Note that as Sae2 sumoylation was strongly increased at 37°C (S1D Fig.), *sae2-K97R* phenotype in the above assays (except Fig. 5E) was examined at this temperature.

qPCR-based resection assay was performed as described [48]. In brief, 150 ng of genomic DNA isolated as above was subjected to restriction enzyme digestion with StyI or mock-digested in a reaction volume of 15 ul. DNA was diluted by addition of 55 ul of ice-cold dH2O. 8.8 ul of the diluted DNA was used for each qPCR reaction in a total volume of 20 ul. Primer sequences are specified in [48]. PCRs were performed using SsoAdvanced Universal SYBR Green Supermix (Bio-Rad) with the Bio-Rad DNA Engine Chromo 4 system and corresponding software (Opticon). All reactions were amplified using the following program: 95°C for 10 min, 40 cycles of (95°C for 15 s, followed by 58°C for 60 s), and melting curve 10 min. Reactions were set up in triplicates for all primer pairs and the resulting average threshold cycle (C_t_) value was used for calculation. The percentage of DNA resected to 0.7 kb in HO-cut DNA was calculated by x = 200/{(1+2^ΔCt^)*f}, where ΔC_t_ = C_t,digestion_−C_t,mock_, and f is the fraction cut by HO as quantified by Southern blot analysis. All C_t_ values were corrected for DNA concentrations by comparing with values for amplification at the *ADH1* locus. For both resection assays, at least two spore clones of each genotype were examined in two or more trials.

### NHEJ assays

The analysis of chromosomal NHEJ levels was performed as previously described [87]. DSB induction was induced for 1.5 h, was performed side-by-side with the resection assay (compare cleavage efficiency at 1.5 h). DSBs were induced in cells that cannot repair the break by HR and rely on NHEJ for repair; thus, NHEJ proficiency can be discerned by comparing the numbers of colonies that survive transient DSB induction. Plasmid-based NHEJ assay was performed by transforming either 1 ng of undigested or 20 ng of BamHI-digested pRS416 plasmid carrying *URA3* into competent cells, and plating on medium lacking uracil. For *yku70*Δ control cells, 100 ng of digested plasmid was used for transformation. Successful NHEJ repair results in ligation of the linearized plasmid and thus growth on -URA medium. Transformation efficiency was calculated as the number of colonies on -URA medium divided by the amount of DNA transformed. NHEJ repair for each genotype was calculated as the ratio of transformation efficiencies of digested to undigested samples. For both NHEJ assays, at least two spore clones of each genotype were examined in two or more independent trials.

### Other methods

Spot assays were performed as described previously [17]. Briefly, log phase cells were diluted 10-fold and spotted onto YPD media with or without CPT or MMS. Plates were incubated at 30°C (Fig. 5C) or 37°C (Figs. 2D, 2G and 3E–3F), and photographed after 24–72 h. At least two spore clones of each genotype were examined in two or more independent trials. Sporulation assay was performed essentially as described [36]. Diploid SK1 cells were grown overnight in YPD medium, washed twice with warm sporulation medium, and left in sporulation medium for 36 h at 30°C. The percentage of sporulated cells was determined by light microscopy.

## Supporting Information

S1 Fig **Sae2 is sumoylated** *in vitro* **and** *in vivo* **, and** *sae2-K97R* **does not affect protein levels.** **A**. Sequence alignment of Sae2 and its orthologs from closely related *Saccharomyces* species at the sumoylation site. The sumoylated lysine of Sae2 is indicated by the red arrow. **B**. Sae2 sumoylation in *E. coli* and validation by Ulp1 treatment. See Materials for details. **C**. Sumoylation of Sae2 does not affect its protein levels. Sae2-K97R (KR) protein levels are similar to wild-type (WT) before (-) or after treatment with CPT, hydroxyurea (HU) or MMS at 37°C. Extracts from *SAE2-TAP* cells exposed to the indicated agents were analyzed by western blotting with antibody recognizing TAP. **D**. Sae2 sumoylation is induced by CPT, MMS or elevated temperature. TAP-tagged Sae2 from cells treated with the indicated agents or elevated temperature was immuno-precipitated and analyzed as in Fig. 1A.
(EPS)

S2 Fig *sae2-K97R* **impairs DSB end resection and increases NHEJ in** *sgs1Δ exo1*Δ** cells.** **A–C**. Formation of end clipping products is impaired by *sae2-K97R*. **A**. Left: Diagram illustrating the resection assay. Induction of the HO endonuclease results in a double strand break at the *MAT*a locus. Resection can be visualized by the disappearance of cut fragment released by HO and digestion with StyI (S). This 0.7 kb cut fragment (double arrowed line below the locus, middle panel) can be visualized with a probe (dark line above the construct) on Southern blots. Right: End clipping was examined in *sgs1*Δ* exo1*Δ cells where extensive resection is blocked. The HO cut fragment (HO cut) and the end clipping fragments below them (bracket) were examined using the indicated probe (left) on Southern blots at indicated time points after HO induction. An example of this analysis is shown with the loading control. **B**. HO cut fragment persists longer in *sae2-K97R* cells. The 0.7 kb cut fragment, i.e. the unprocessed fragment from (A) and two more trails of similar experiments was quantified for each time point, normalized to the loading control and compared to the value at 30 min. Note that HO cleavage efficiency was taken into account at each time point. **C**. *sae2-K97R* reduces the levels of end clipping products. The percentage of end clipping products within total cut fragment was calculated for each genotype based on at least three experiments. The averages and standard deviations at each time point are shown. The difference between the two strains for each time point is statistically significant (p<0.05, asterisk). **D**. Lack of Sae2 sumoylation results in higher NHEJ levels in the *sgs1*Δ* exo1*Δ background. Chromosomal NHEJ was assayed using the same construct as in A. As the homologous repair template is absent, cells can survive transient HO induction only if NHEJ seals the break. Thus, survival percentage is a readout of NHEJ efficiency. Asterisks indicate statistically significant differences (p<0.05, asterisk).
(EPS)

S3 Fig **Reduction of MRX sumoylation impairs DSB end resection.** **A–B**. Resection efficiency is reduced in *YKU70-UD* compared to YKU70-UD* cells. **A**. A representative result of resection assay as in S2A Fig. is shown. **B**. Quantification of the ratio of unprocessed HO cut fragment in YKU70-UD* to that in *YKU70-UD* is plotted based on at least three independent trials. Asterisks indicate statistically significant differences (p<0.05). Three spore clones of each genotype were examined.
(EPS)

S4 Fig **Sumoylation time course and aggregation potential profile of Sae2.** **A**. Sae2 sumoylation was examined in the experimental conditions for testing solubility of Sae2. Time course was performed as in Fig. 4 and sumoylation levels were examined as in Fig. 1A. **B**. Aggregation potential profile of Sae2 with sumoylation site shown in red and phosphorylation sites indicated in purple. Aggregation potential for a protein was calculated by AGGRESCAN [73], which is based on amino acid measurements from *in vivo* experiments and assumes that short and specific stretches of amino acids regulate protein aggregation.
(EPS)
